# Olfactory neurogenesis plays different parts at successive stages of life, implications for mental health

**DOI:** 10.3389/fncir.2024.1467203

**Published:** 2024-08-08

**Authors:** Jules Dejou, Nathalie Mandairon, Anne Didier

**Affiliations:** ^1^INSERM, U1028; CNRS, UMR5292; Lyon Neuroscience Research Center, Neuroplasticity and Neuropathology of Olfactory Perception Team, Lyon, France; ^2^Institut Universitaire de France, Paris, France

**Keywords:** neurogenesis, olfactory bulb, mouse, development, learning, memory, mental disorders

## Abstract

The olfactory bulb is a unique site of continuous neurogenesis, primarily generating inhibitory interneurons, a process that begins at birth and extends through infancy and adulthood. This review examines the characteristics of olfactory bulb neurogenesis, focusing on granule cells, the most numerous interneurons, and how their age and maturation affect their function. Adult-born granule cells, while immature, contribute to the experience-dependent plasticity of the olfactory circuit by enabling structural and functional synaptic changes. In contrast, granule cells born early in life form the foundational elements of the olfactory bulb circuit, potentially facilitating innate olfactory information processing. The implications of these neonatal cells on early life olfactory memory and their impact on adult perception, particularly in response to aversive events and susceptibility to emotional disorders, warrant further investigation.

## Introduction

Inhibitory neurons of the olfactory bulb (OB), periglomerular cells and granule cells (GCs), which shape the output message of the OB (Nagayama et al., 2014), are formed throughout life, from pre-and neonatal periods, to adulthood and senescence. Although forming a continuum across life, bulbar neurogenesis characteristics evolve with aging. Bulbar neurogenesis relies on stem cells and amplifying progenitors sitting in the proliferative subventricular zone (SVZ). They give birth to neuroblasts, proceeding along the rostral migratory stream to the core of the OB before differentiating and integrating the granule (GCL) and periglomerular cell layers of the OB (Luskin, 1993; Alvarez-Buylla and García-Verdugo, 2002; Winner et al., 2002). In the early postnatal period, an additional neurogenic zone in the OB core produces over 50% of new interneurons, before this zone gradually ceases to be a proliferative niche (Lemasson et al., 2005). Distinctive features of adult-born versus neonatal neurons include their rate of production, pattern of integration and final location in the OB as well as sensitivity to environment. In this review we emphasize recent advances that established the role of adult neurogenesis in experience-dependent plasticity of the OB circuits and enabled distinguishing bulbar interneurons born at different stages of life. These advances emerged mostly from rodent studies and focused on the GCs, the most abundant neuronal population targeted by neurogenesis. We also briefly examine the role of early neurogenesis in vulnerability to mental disorders.

## Descriptive features of olfactory neurogenesis across the life span

### The rate of neurogenesis

The rate of neurogenesis in the OB, i.e., of new neurons formed, is the highest between P0 until P14, corresponding to the ontogeny of approximatively 75% of GCs and relies on high proliferation and virtually no cell death (Lemasson et al., 2005; Sakamoto et al., 2014). As the animal reaches sexual maturity (2-month-old), GCs are more prone to death (Lemasson et al., 2005; Sakamoto et al., 2014) and by 10 months, proliferation is decreased by 50 to 75% in the SVZ (Rey et al., 2012; Shook et al., 2012) accounting for the reduction in the number of new GCs formed in the OB. In the last part of life (18-month-old), proliferation in the SVZ is not further reduced but survival of the newly formed GCs decreases, leading to the lowest rate of neurogenesis in life (senescent rate) (Rey et al., 2012). Thus, the rate of neurogenesis in the mouse OB distinguishes between neonatal, juvenile, adult and senescent stages with transitions whose gradual or abrupt nature is not clearly documented.

### Neuronal addition versus replacement

There is a consensus that neonatal neurogenesis follows an addition mode: the newborn GCs integrate the developing OB, enabling its growth. This is supported by the lack of cell death reported for these neonatal neurons (Lemasson et al., 2005; Platel et al., 2019). In the adult stage, the data are more contradictory. Several studies labeled dividing cells by thymidine analogs (such as 5-Bromo-2′-deoxyuridine, BrdU) and reported a loss of up to 50% of adult-born neurons within 4 to 6 weeks after their birth (Lemasson et al., 2005; Mandairon et al., 2006b). This suggests that new GCs compete for survival within the OB network but that not all of them succeed. In line with this, genetic tagging allowing continuous follow-up of newly formed GCs revealed that adult-born neurons end up forming at least 60% of the GCs in 18-month-old mice, without increasing neuronal density (Imayoshi et al., 2008). This supports the conclusion that in adults, neurogenesis follows a replacement mode where newborn neurons replace dying ones. However, Platel et al. (2019), using bi-photon time-lapse observations, reported a lack of adult-born GCs death supporting an addition mode in adult as in the developing OB. BrdU toxicity was brought forward to explain previous reports of cell death affecting adult-born GCs. Noteworthy, studies using viral vectors or non-toxic doses of BrdU did detect differences in survival between neonatal and adult-born neurons (Lemasson et al., 2005; Sakamoto et al., 2014). Interestingly, due to technical constraints (i.e., 600 μm depth limitation, labeling of dorsal SVZ neuroblasts, targeting the superficial GCL (De Chevigny et al., 2012)), bi-photon analysis captures GCs in the superficial GCL which is preferentially populated by neonatal GCs that do not show cell death in BrdU studies (Lemasson et al., 2005). This suggests that superficial GCs may have different survival properties compared to those targeting the deeper GCL. Thus, the survival rate of adult-born neurons may depend both on the age of the animals and the depth of the GCs in the GCL.

In the neuron’s replacement framework, many questions remain such as which GCs are selected to be renewed and what specific parts they play versus the non-renewed ones.

### Synaptic maturation patterns

**Figure 1:**
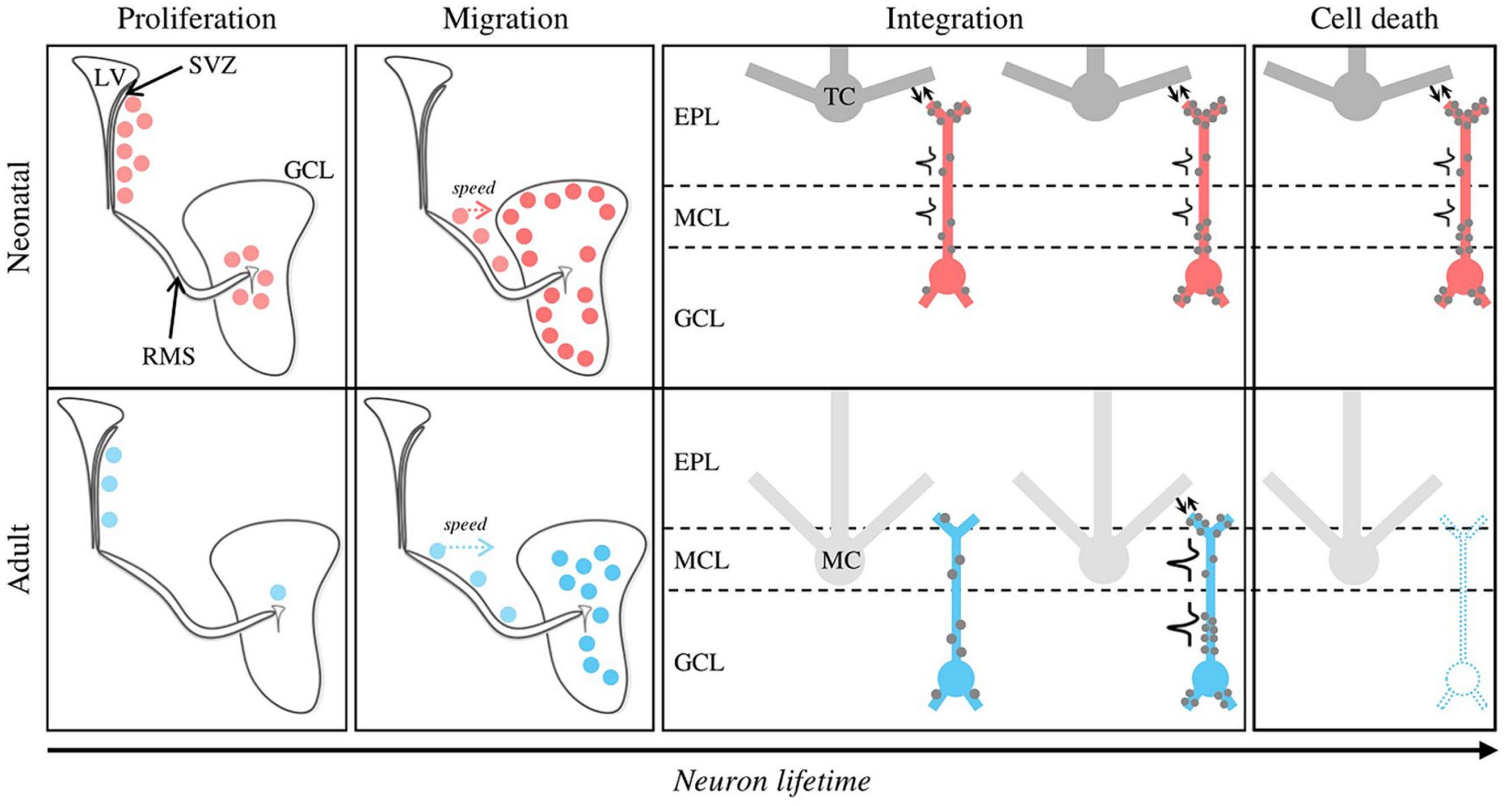
Distinctive features of neonatal versus adult postnatal neurogenesis. Newly formed GCs at neonatal and adult stages are represented by red and blue dots, respectively. *Proliferation mode*. Neonatal GCs originate from the SVZ and the OB core in the same proportion, whereas adult neurogenesis is almost exclusively dependent on the SVZ. The neurogenesis rate peaks at neonatal stage. *Migration*. Although the distance between the SVZ and the OB is greater at the adult stage, adult-born GCs migrate to the OB faster compared to their neonatal counterparts. Neonatal and adult-born GCs preferentially join the superficial and deep regions of the GCL, respectively. *Structural integration*. Due to their differential targeting inside the GCL, neonatal and adult-born GCs preferentially contact tufted and mitral cells, respectively. For neonatal GCs, inputs (gray dots) and outputs (reciprocal synapses, bidirectional arrows) are simultaneously established on the proximal and distal dendrites, then on the basal dendrites. Adult GCs follow a “first listen then act” mode: inputs are established on the proximal and basal dendrites, then form inputs-outputs through dendro-dendritic synapses. *Functional integration*. Neonatal GCs can emit action potentials shortly after the end of their migration, which is not the case for adult-born GCs. *Cell death*. Neonatal GCs have a much higher survival rate than adult-born GCs, which undergo massive turnover (~50%, dotted GC). EPL, External Plexiform Layer; GCL, Granule Cell Layer; LV, Lateral Ventricle; MCL, Mitral Cell Layer; RMS, Rostral Migratory Stream; SVZ, Subventricular Zone.

The dynamic integration of newborn GCs shows remarkable differences depending on when they were born. Neonatal GCs need more time to migrate from the SVZ to the OB than their adult counterparts (Lemasson et al., 2005). Then, in the adult brain, it takes about a month for a neuroblast arriving in the OB to acquire the anatomy and connectivity of a fully mature GC (Carleton et al., 2003; Kelsch et al., 2008; Tufo et al., 2022) according to a “listen then act” mode where synaptic inputs develop before outputs. More specifically, inputs originating mainly from centrifugal axons contact first basal dendrites of adult-born GCs while the later developing output is via dendrodendritic synapses in the external plexiform layer of the OB (Whitman and Greer, 2007). Regarding these dendrodendritic reciprocal synapses, it appears that the excitatory mitral-to-granule part forms before the inhibitory granule-to-mitral part (Hinds and Hinds, 1976). In contrast, synaptic inputs and outputs occur simultaneously in neonatal GCs, resulting in the ability of these neurons to emit action potentials shortly after their arrival in the OB (Kelsch et al., 2008; Breton-Provencher and Saghatelyan, 2012). Selective genetic alteration of GABA_A_ receptors in migrating neuroblasts or immature GCs delays their synaptic integration and morphological maturation (Pallotto et al., 2012). This early sensitivity to GABA could explain how olfactory stimulation promotes the integration of very young (1–2 week-old) adult-born GCs (Mandairon et al., 2006a), since olfactory inputs would activate inhibitory GCs and thus increase GABA release. As they integrate in the bulbar circuits, newborn GCs become capable of responding to olfactory stimulation. The younger the neurons, the more excitable they are, and long-term potentiation is easier to induce in 2- to 3-week-old adult-born neurons than in older ones (Magavi, 2005; Gao and Strowbridge, 2009; Nissant et al., 2009). These peculiar features of maturing adult-born GCs suggest that they play selective parts in odor-induced circuit plasticity (Figure 1).

At mid-age (10-12-month-old), the number of adult-born GCs is reduced to about one third of that observed in 2-month-old animals but their dendritic and synaptic equipment is similar (Greco-Vuilloud et al., 2022) and little is known regarding integration patterns of GCs born in the aged brain (18-month-old).

## Functional implications of bulbar neurogenesis

### Adult OB neurogenesis contributes to olfactory learning in a task-specific manner

Olfactory perception and MCs responses to odorants evolve with experience and learning (Kay and Laurent, 1999; Doucette and Restrepo, 2008; Gschwend et al., 2015). The contribution of permanent neurogenesis to this plasticity has been addressed in adult mice, using a variety of olfactory tasks, such as olfactory enrichment, associative or perceptual olfactory discrimination learning. All increase the number of adult-born GCs in the OB by improving their survival (Rochefort et al., 2002; Alonso et al., 2006; Mouret et al., 2008; Belnoue et al., 2011; Mandairon et al., 2011). Olfactory experience also increases the proportion of adult-born GCs responding to a learned odorant (Belnoue et al., 2011; Sultan et al., 2011; Moreno et al., 2012). On the contrary, ablation of neurogenesis by antimitotic drugs (Breton-Provencher et al., 2009; Moreno et al., 2009) or SVZ irradiation (Lazarini et al., 2009) prevents discrimination learning. Optogenetic inhibition (Forest et al., 2019a) or activation of adult-born GCs (Alonso et al., 2006; Grelat et al., 2018) respectively impairs or accelerates learning performances. This establishes a causal relationship between the adult-born GCs’ activity and olfactory learning.

At the cellular level, olfactory learning or deprivation acts on adult-born GCs notably through structural plasticity. Indeed, both can lead to addition, elimination or relocation of dendritic spines at both long (hours-days) and short (minutes) timescales (Saghatelyan et al., 2005; Kelsch et al., 2008; Livneh and Mizrahi, 2011; Lepousez et al., 2014; Breton-Provencher et al., 2016; Sailor et al., 2016; Hardy and Saghatelyan, 2017; Mandairon et al., 2018; Forest et al., 2019a; Ferreira et al., 2024). Some molecular actors of adult-born GCs structural plasticity have been identified such as Npas4, an activity-dependent protein promoting pruning in response to sensory inputs (Belvindrah et al., 2011; Yoshihara et al., 2014) or FMRP, a regulator protein of local translation in dendrites (Daroles et al., 2016; Messaoudi et al., 2024).

The plasticity of adult-born neurons can be task-specific. In the context of olfactory perceptual learning in mice, in which two perceptually close odorants are learnt to be discriminated by passive exposure, spine density was increased on apical dendrites of adult-born GCs as was MCs inhibition, promoting discrimination through pattern separation. In contrast, when discrimination is acquired by olfactory associative learning in which the two perceptually close odorants are learnt to be discriminated by rewarding one odorant of the pair, spine density on adult-born neurons was reduced and activation of MCs increased, thereby strengthening of MCs’ responses to the reinforced odorant to enable discrimination (Mandairon et al., 2018). As another example of the task-specificity of adult-born GCs involvement, a non-operant paradigm of olfactory associative conditioning showed no dependence on adult-born neurons (Imayoshi et al., 2008; Breton-Provencher et al., 2009) unlike operant conditioning (Lazarini et al., 2009; Mandairon et al., 2011; Sultan et al., 2011). Interestingly, odor fear conditioning, a non-operant learning associating an odorant to an electric shock, does require adult neurogenesis (Valley, 2009). Thus, task engagement, motivation and context are key factors in the contribution of new GCs to the plasticity of the bulbar network related to experience and learning, which remains difficult to disentangle.

Taken together, these data indicate that in young adult mice, different forms of olfactory experience non-specifically increase the survival of adult-born GCs, while their synaptic integration pattern appears to be finely tuned to the task, allowing individuals to adapt to the needs of a changing olfactory environment.

### Implication of newly formed neurons depending on their birthdate and maturation level

A question raised by permanent neurogenesis concerns the specificity of the role of newborn GCs according to the age of the brain in which they are born and their own level of maturation. This dual temporality makes this a complex question to address experimentally. Neonatal GCs increase their dendritic spine density until day 26 (Kelsch et al., 2008). During this period of maturation, they are prone to exhibit LTP (Gao and Strowbridge, 2009). But how immature neonatal GCs contribute to early-life olfactory perception and odor-guided behaviors is poorly understood. It has been suggested that they represent the building blocks of OB functions linked to vital, innate, enduring, and inflexible olfactory behaviors (Lemasson et al., 2005; Alonso et al., 2012; Muthusamy et al., 2017; Grelat et al., 2018). The evidence in support of this view is indirect, based in particular on the different local circuits mediated by the GCs located in the superficial GCL, which houses the majority of GCs born in the neonatal period, and the deeper GCs, the main target of adult-born neurons and neuronal renewal. Superficial GCs mainly come into contact with tufted cells (TCs), while deeper GCs are more in contact with MCs. TCs and MCs exhibit different olfactory response dynamics and cortical projections (Nagayama et al., 2010; Miyamichi et al., 2011; Fukunaga et al., 2012; Igarashi et al., 2012) leading to the hypothesis of the existence of two parallel circuits: neonatal GCs and TCs involved in odor detection and operational at the early stage of development and adult-born GCs and MCs, operating from the first weeks of life, handling more complex olfactory demands. However, this segregation may be too simplistic, as even if they have preferential targeting in the GCL, GCs may target deep or superficial parts regardless of their date of birth, and their location in the GCL does not completely condition their local connectivity (Yoshihara et al., 2012; Takahashi et al., 2018; Tsuboi, 2020).

The functional role of adult-born GCs may also depend on their level of maturation. They are better rescued from cell death by olfactory learning (Alonso et al., 2006; Mandairon et al., 2006a; Mouret et al., 2008; Belnoue et al., 2011) and activated by olfactory inputs in their immature state (14- to 28-day-old) (Magavi, 2005; Whitman and Greer, 2007; Nissant et al., 2009; Breton-Provencher and Saghatelyan, 2012). Immature adult-born GCs also exhibit learning-induced structural plasticity not shown by mature neonatal GCs (Breton-Provencher et al., 2016; Forest et al., 2019a). Their optogenetic inhibition impaired learned discrimination while the same photo-inhibition applied on mature neonatal GCs impaired only complex perceptual discrimination learning (Forest et al., 2019a). Interestingly, photo-activation of mature adult-born GCs (10- to 12-week-old) can accelerate associative olfactory learnings (Alonso et al., 2012; Grelat et al., 2018; Bragado Alonso et al., 2019).

From 10-month-old, olfactory enrichment no longer increases the survival of immature GCs (Rey et al., 2012). At the same time, they show impaired structural plasticity in response to olfactory learning and lower performances (Greco-Vuilloud et al., 2022). Such impairments of neurogenesis may, at least in part underlie olfactory cognitive decline, prompting exploration of ways to maintain a higher level of olfactory plasticity with age (Terrier et al., 2024). In the aged brain, new neurons become very rare, questioning their functional significance.

**Figure 2:**
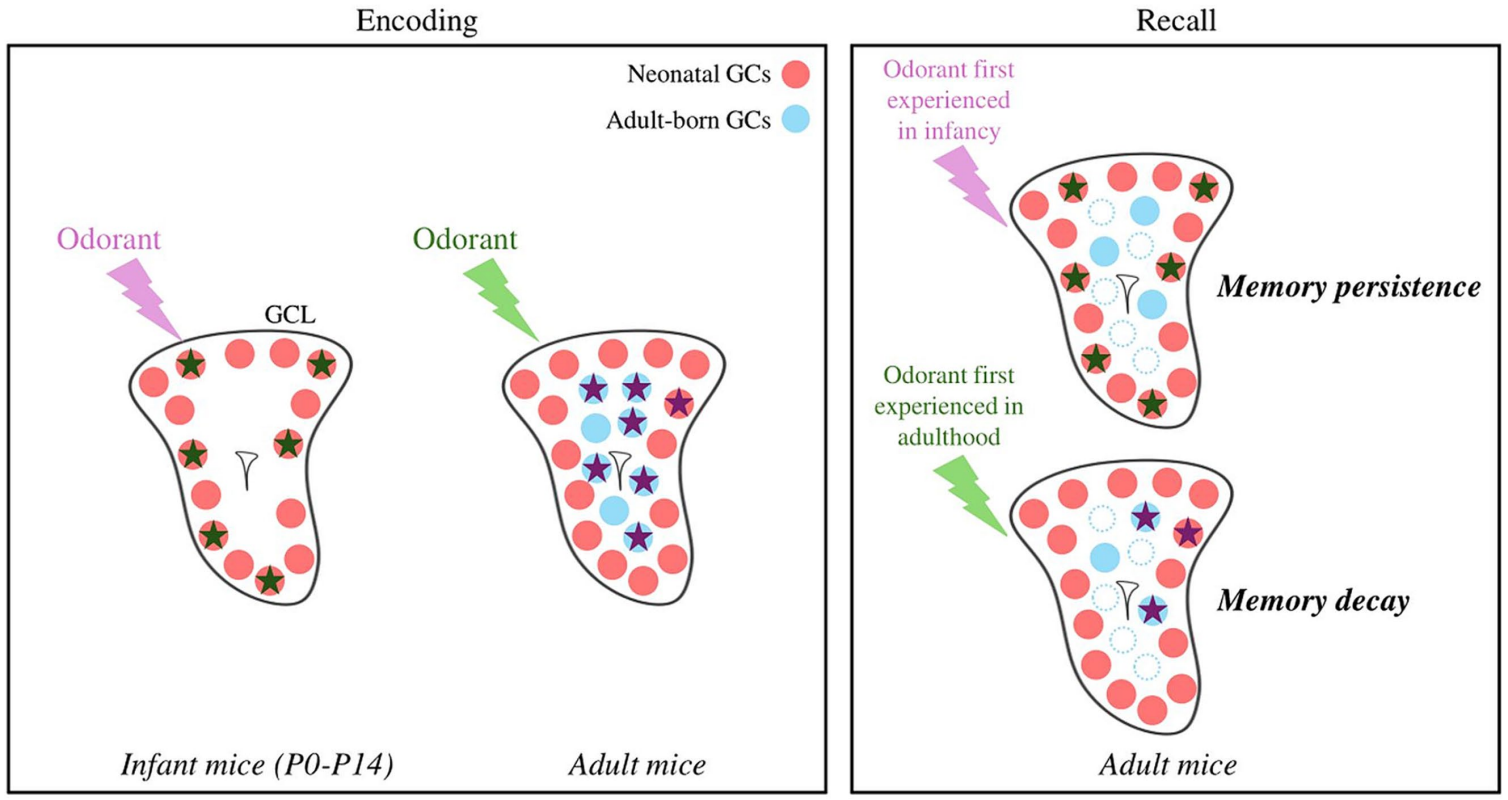
Hypothetical model of odor and memory processing by newly formed GCs according to the age of the animal. GCs activated in response to an odorant are represented by stars. An odorant processed at the infant stage is encoded by neonatal GCs, which show little cell death. The local network is reactivated by the odorant during recall, allowing the persistence of the memory. An odorant processed at the adult stage is encoded by adult-born GCs, showing greater renewal (blue dotted dots). The local network is only partially reactivated, possibly explaining the decay of the olfactory memory. Note that an adult olfactory learning can also recruits neonatal GCs. GCL, Granule Cell Layer; GCs, Granule Cells.

To conclude, GCs born in the adult brain play a decisive and specific role in experience-induced plasticity during their immature state. GCs born early after birth are essential for assuming innate olfactory behaviors, not least because they are the only ones present at birth. In addition, and given their high survival rate, it’s tempting to speculate that they might retain a lifelong memory of early olfactory events that could escape the fading caused by neuronal turnover (Figure 2) (Frankland et al., 2013; Epp et al., 2016; Forest et al., 2019b). Finally, GCs differ not only based on their location in the GCL but also on their origin in the SVZ (Merkle et al., 2007, 2014), morphology, transcriptome (for recent studies see Takahashi et al., 2016, 2018; Malvaut et al., 2017; Hardy et al., 2018; Tsuboi, 2020; Fernández Acosta et al., 2022) but this diversity has not yet been considered in functional studies of adult bulbar neurogenesis.

## Role of centrifugal feedbacks

Regardless of the age of the animal, the integration of newborn GCs in the OB is context-dependent, suggesting that it may be controlled by top-down inputs. The OB sends projections to olfactory cortices (Igarashi et al., 2012; Hanson et al., 2020) which in turn, send information to the OB (Luskin and Price, 1983; Shipley and Adamek, 1984; Padmanabhan et al., 2018; In’t Zandt et al., 2019). Interestingly, cortical fibers establish synaptic contacts with adult-born GCs (Arenkiel et al., 2011; Deshpande et al., 2013; De La Rosa-Prieto et al., 2015) whose density is increased by olfactory learning (Lepousez et al., 2014) and activation can induce LTP (Nissant et al., 2009). Recently, the chemogenetic stimulation of the anterior olfactory nucleus glutamatergic feedbacks to the OB was shown to reduce adult-born GCs survival (Libbrecht et al., 2021). In contrast, the GABAergic inputs from the horizontal band of Broca area (HDB) favor their survival (Hanson et al., 2020). This study also revealed that GABAergic contacts from the HDB on newly formed GCs are denser in the superficial than in the deep part of the GCL, and thus may have different impact neonatal versus adult-born GCs. The centrifugal inputs develop early in life, from birth or the immediate post-natal period (piriform cortex, anterior olfactory nucleus), or from the second week of life (cortical amygdala, lateral entorhinal cortex) suggesting that the top-down influence on newly formed cells occurs from birth and evolves as a function of the individual’s age (Kostka and Bitzenhofer, 2022).

The OB is also the target of the noradrenergic, cholinergic (Mandairon and Linster, 2009; Mouret et al., 2009; Schilit Nitenson et al., 2019; Zhou et al., 2022) and serotoninergic (Banasr et al., 2004; García-González et al., 2017) neuromodulatory systems whose influence is critical to olfactory learning. In adults, the action of the noradrenergic and cholinergic systems on newborn GCs are required for the perceptual olfactory learning to occur (Moreno et al., 2012; Schilit Nitenson et al., 2019). Thus, it seems that these two systems have synergic effect enabling the integration of newborn GCs in the OB circuitry. In associative learning, inhibiting noradrenergic actions did not affect behavioral responses nor neurogenesis rate (Vinera et al., 2015), suggesting task-specificity of the neuromodulatory systems actions. The noradrenergic fibers coming from the *Locus Coeruleus* are present at birth. They are inhibitory on GCs at neonatal stages (Pandipati and Schoppa, 2012) and play a role in neonatal imprinting behaviors (Moriceau and Sullivan, 2004). Regarding acetylcholine, the time course of development of its contacts with newly-formed GCs and function are largely unknown.

## Implication for mental health

Neuropsychiatric conditions like depression, anxiety, schizophrenia or borderline personality disorder often stem from early life stress (Anda et al., 2006; Pietrek et al., 2013; Youssef et al., 2019; Lippard and Nemeroff, 2020; McKay et al., 2022), and are accompanied by olfactory dysfunctions (Naudin et al., 2012; Kazour et al., 2017; Kamath et al., 2018; Crow et al., 2020; Li et al., 2020; Athanassi et al., 2021; Chen et al., 2021; Marin et al., 2023). Adult neurogenesis has been shown to play a part in animal models of depression (Siopi et al., 2016; Ren et al., 2021) but translation to humans is uncertain because the existence of adult neurogenesis in humans is controversial (Lucassen et al., 2020; Duque et al., 2022; Alshebib et al., 2023; Alonso et al., 2024). In contrast, could alteration of early life neurogenesis play a part in the vulnerability to psychiatric diseases induced by early life aversive events? Indeed, neurogenesis during childhood in humans is less prone to debate (Sanai et al., 2011; Dennis et al., 2016; Mathews et al., 2017; Cipriani et al., 2018; Sorrells et al., 2018). The large overlap in neural structures mediating emotional behavior and processing of olfactory signals suggests that the OB could contribute to emotional disturbances (Kontaris et al., 2020; Bhattarai et al., 2022). In support to this hypothesis, early life stress reduced developmental olfactory neurogenesis (Naninck et al., 2015). Thus, altering the neurogenesis at this developmental step could durably alter OB structure and function and contribute to long-lasting olfactory deficits and alteration of emotional behavior (Athanassi et al., 2021; Marin et al., 2023).

## Author contributions

JD: Writing – original draft, Writing – review & editing. NM: Writing – original draft, Writing – review & editing. AD: Writing – original draft, Writing – review & editing.
